# PRORED: a hybrid transformer framework with progressive refinement decoding for segmenting dynamic speech MRI

**DOI:** 10.1093/bjrai/ubaf020

**Published:** 2025-12-29

**Authors:** Ying He, Qianni Zhang, Marc E Miquel

**Affiliations:** School of Electronic Engineering and Computer Science, Queen Mary University of London, London E1 4NS, United Kingdom; Clinical Physics, Barts Health NHS Trust, London EC1A 7BE, United Kingdom; School of Electronic Engineering and Computer Science, Queen Mary University of London, London E1 4NS, United Kingdom; Clinical Physics, Barts Health NHS Trust, London EC1A 7BE, United Kingdom; Magnetic Resonance and Non-ionising Radiation Physics, Medical Physics and Clinical Engineering, Guy's and St Thomas' NHS Foundation Trust, London SE1 7EH, United Kingdom; School of Biomedical Engineering and Imaging Sciences, King’s College London, London SE1 7EH, United Kingdom

**Keywords:** speech MRI, image segmentation, vision transformer, feature refinement

## Abstract

**Objectives:**

Dynamic MRI of the upper vocal tract is increasingly used to study speech. Image segmentation is often required to analyse the organs of speech; however, manual segmentation is labour intensive and time consuming and automatic methods are being developed. In this paper, a new hybrid transformer network is proposed for such task.

**Methods:**

We introduce a deep learning-based decoder model termed “Progressively Refinement Decoding (PRORED).” This model incorporates a directional field (DF) module designed to capture the contour details of features. The acquired contour information is leveraged to refine the boundaries both between and within classes. By integrating the DF module at different stages of the decoder, features are enhanced progressively, ensuring a more detailed and accurate segmentation.

**Results:**

Our model is evaluated using a publicly accessible speech MRI dataset and a cardiac dataset. The metrics employed are the Dice coefficient and the Hausdorff distance. Results indicate that our model attains an average Dice coefficient of 97.78% and a Hausdorff distance of 6.84 mm. Additionally, our network was able to identify closure patterns more efficiently than the baseline network and previously published work. In addition, the model was also evaluated on a cardiac dataset, and achieved 91.90% dice score.

**Conclusions:**

The proposed model leads to a more accurate segmentation of speech MRI data and in particular allows for a better velopharyngeal closure study. The proposed model was also evaluated on a cardiac dataset and achieved competitive performance, showing its strong generalizability.

**Advances in knowledge:**

First model that utilizes vision transformer and progressive refinement decoder to segment dynamic speech MRI.

## Introduction

Dynamic Magnetic Resonance Imaging of the upper vocal tract is increasingly used to study swallowing and speech, both for linguistic research and in a clinical context.^1^^,^^2^ Swallowing function is used in numerous conditions and is clinically carried out using video fluoroscopy. Despite the limitations linked to the position in the scanner, dynamic MRI clearly demonstrated its potential thanks to its better image contrast without ionizing radiation.^3–6^ However, it is in the study of speech that dynamic MRI is becoming the method of choice as it provides information on the shape, size, motion, and position of the vocal tract and articulators.^1^^,^^7^ In phonetics and linguistics, it has helped increasing our understanding of speech production and learning processes in various languages.^8–13^ Clinically, Dynamic MRI has been mainly used to study apraxic speech, speech following tongue surgery^14–16^ and velopharyngeal insufficiency (VPI) in particular in people with repaired cleft palate.^8^^,^^17–20^ Therefore, it is crucial to have a well segmented soft palate and pharyngeal wall in speech MRI. Speech analysis is most commonly carried out on a temporal series of 2 dimensional (2D) magnetic resonance (MR) images. Although early studies, might have been using visual assessment or simple region of interest, most recent studies have used segmentations of the articulators or their surface.^7^ In linguistics, it allows for the analysis of vocal tract shaping^21^ or the shape of the tongue^22^ while clinically it can facilitate the analysis of the shape of the soft palate^23^ and velopharyngeal closure events.^24^ Recently, segmentation was used as a first step before registration, hence helping to calculate the motion of the different articulators.^25^ Manual segmentation is time consuming, especially in dynamic datasets containing upwards of hundred frames, consequently, there is a growing interest in developing automatic segmentation. Skordilis et al. segmented the airway using a semi-automated approach based on the seed growing algorithm.^26^ Bresch et al.^27^ first described an unsupervised technique for segmenting the upper airway, employing an anatomically informed object model to process a series of real-time MRI images. Similarly, Silva et al.^28^ introduced an unsupervised segmentation method for vocal tract images from upper airway real-time MRI, based on an active appearance model. Following the success of the deep learning paradigm in medical image analysis, a number of methods for automatically segmenting the airway in MRI have been developed. Valliappan et al. segmented the air tissue boundary using Segnet,^29^ Lipeng et al. utilize CNN to segment the upper airway,^30^ and Sublin et al. employed UNet to automatically segment the vocal tract airway.^31^ To segment distinct groups of articulators and vocal tract in speech MR images, Ruthven et al. developed a model similar to the original UNet,^24^ in contrast to,^26^^,^^29^^,^^31^ which primarily focus on segmentation of the airway. Building on,^24^ Peplinski^32^ further trained an FCN network, specifically focusing on images cropped around the vocal tract. Most existing methods for segmenting the vocal tract and speech organs are based on CNN. However, CNNs often exhibit limitations in modelling long-range spatial dependencies, restricting the model’s ability to comprehend broader contexts, and thereby affecting the accuracy of segmentation outcomes. In our work, we leverage the Vision Transformer’s ability to capture long-range spatial dependencies.^33^ Given the importance of accurately capturing the soft palate’s detailed shape, we propose a progressive refinement decoder that enhances segmentation accuracy by progressively refining predictions at multiple scales in the decoder layers.

Recently, Vision Transformers (ViTs) have increasingly garnered attention in the field of computer vision, since their introduction for image classification.^33^ ViTs represent a paradigm shift from traditional CNNs by employing a structure that divides images into fixed-size patches or tokens, which are then embedded with positional information and processed through transformer encoders and a Multilayer Perceptron (MLP). Although groundbreaking, this architecture is bound by its inherent limitations, in particular, its simple tokenization fails to efficiently capture local structures like edges, resulting in limited feature richness and inefficient training.^34^ To solve feature richness issues, the Tokens-To-Token Vision Transformer (T2T) was developed^34^ while computational challenges were addressed by Swin-Transformer and SegFormer that adopt hierarchical transformer.^35^ In addition, DeiT explores data-efficient training strategies, focusing on minimizing the computational cost for ViT.^36^ Our primary clinical interest is the study of closure pattern and VPI (the absence of velopharyngeal closure). This relies on having accurate segmentation of the soft palate and the post-pharyngeal wall. As the soft palate is a small organ, we propose a transformer-based model featuring a hybrid encoder that integrates both convolutional and transformer layers, enabling the capture of both local and global contexts effectively. Given the challenges presented by the similar textures of classes and the small size of the soft palate in speech MRI images, we introduce a novel Progressive Refinement Decoder named PRORED. The decoder progressively refines the multiscale features at the upsampling stages, enhancing the distinction between class boundaries and reinforcing the similarities within classes. The fusion of a hybrid encoder with PRORED empowers the model to grasp a more holistic context, significantly diminishing confusion at class boundaries. The proposed model is tested on the publicly available Speech MRI dataset that also contains associated ground-truth segmentation and closure pattern diagrams. As the primary clinical interest of our group is the study of speech articulator motion and velopharyngeal closure, the proposed model is first tested on a publicly available Speech MRI dataset that also contains associated ground-truth segmentation and closure pattern diagrams. The segmentation of the articulator is a necessary first step in analysing automatically or semi-automatically closure patterns^24^ and soft palate shape^23^ and rise time,^23^ however, it is time consuming if done manually. Although, deep learning models have shown great potential in medical image analysis they still require large amounts of annotated data. Consequently, the proposed model could be used to (1) allow segmentation to address automatic analysis and (2) be used to annotate other rt-MRI speech data sets that do not provide ground truth(GT) segmentation (eg,^37^^,^^38^) thus assisting clinicians in annotating additional data and speeding up and streamlining the annotation process. Finally, to start assessing the generalizability of the proposed network, it is also tested on a cardiac MRI dataset.^39^

## Methods

### Overall architecture

**Figure 1.**
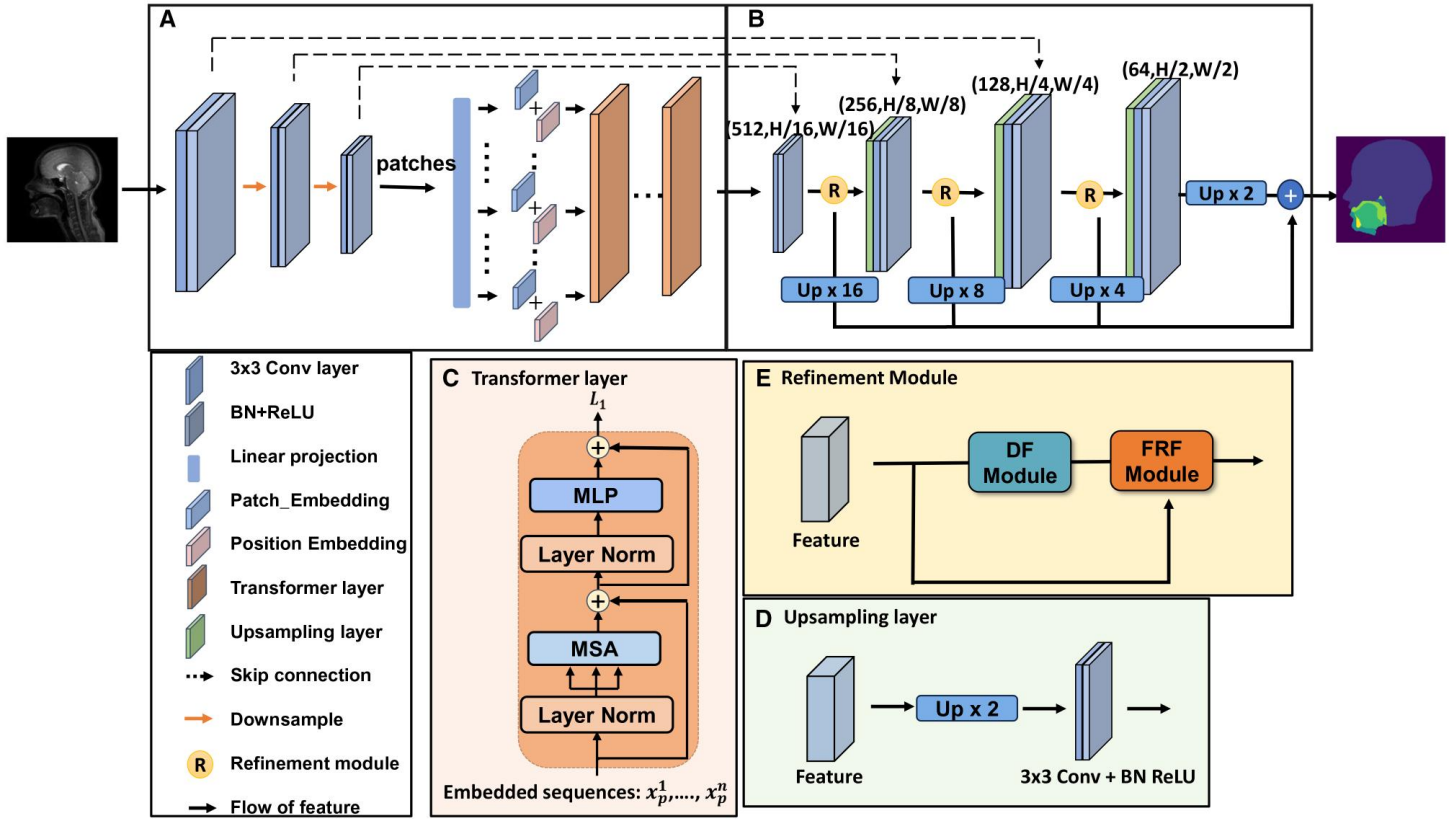
Overall architecture of the proposed model consisting of a hybrid encoder (A) and a PRORED (B). Details of the (C) transformer layer, (D) up-sampling layer, and (E) refinement module are also shown.

The model shown in Figure 1, has 2 key components: a hybrid encoder and PRORED. The hybrid encoder blends CNN and transformer to capture both global and spatial feature dependencies.^40^ PRORED employs up-sampling layers and skip connections to restore feature resolution and information lost during compression, alongside refinement modules that sharpen feature maps’ boundaries. Ultimately, the final segmentation is obtained by merging the refined features in the decoder.

### Encoder

The hybrid encoder combines the strengths of the convolutional and transformer layers. The convolutional layers are used in our encoder to extract high-resolution information from the input image. The high-resolution features are then further encoded using a transformer to extract global context.

Assuming an image of dimension H × W × C is given. The image features are encoded directly by three 3×3 convolutional layers with max-pooling between the layers. Each convolutional layer is followed by a batch normalization and a ReLU activation layer. Then, the encoded features are tokenized by flattening it into a series of 2D patches $\{x_{p}^{i}\in R^{P\times P\times C}|i=1,\ldots ,N\},$ where each patch is of size P×P and total number of the image patches is *N* = $\frac{HW}{P^{2}}$. After that, the patches are transformed into a *D*-dimensional latent space via a trainable linear projection *E*, where $E\in R^{(P\times P\times C)\times D}$. To uphold spatial details, specific position embeddings $E_{\text{pos}}$, where $E_{\text{pos}}\in R^{N\times D}$ are introduced and added to the patch embeddings, therefore, the input of the transformer layer can be written as:


$$z_{0}=[x_{p}^{1}E;x_{p}^{2}E;\ldots ;x_{p}^{N}E]+E_{\text{pos}.}$$

The transformer layer, as shown in Figure 1C, is made up of n layers of multihead self-attention and a MLP block. The output of the $l_{\text{th}}$ layer can be represented as:


$$\begin{array}{cc} z_{l}^{{}^{\prime }} & =\text{MSA}(\text{LN}(z_{l-1}))+z_{l-1},\\ z_{l} & =\text{MLP}(\text{LN}(z_{l}^{{}^{\prime }})+z_{l}^{{}^{\prime }}, \end{array}$$

where the LN(·) denotes the layer normalization operator and $z_{l}$ is the image representation after encoding by the transformer layer.

The image representation from the transformer layer has the shape of $\frac{HW}{P^{2}}\times D$. It is then reshaped to the shape of $\frac{H}{P}\times \frac{W}{P}\times D$, where *P* is the shape of each small patch and set to 16.

### Progressive refinement decoder

The final output from the transformer’s last layer is altered and enhanced by PRORED to improve feature clarity. PRORED incorporates skip connections, up-sampling, and modules for refinement to gradually enhance image resolution. It employs a bilinear up-sampling method followed by a convolutional layer with a 3-kernel size, accompanied by batch normalization and a ReLU activation layer. Skip connections are used to blend encoded and up-sampled features, helping to restore detail that was lost during the encoding process. After each skip connection, directional field (DF) maps refine the features, sharpening the delineation of class boundaries. In the final step, these polished, multi-scale feature maps pass through a convolution, resulting in a segmentation map with a 7-channel output, each channel representing 1 of the 7 classes in the speech MRI dataset.

#### Refinement module

The features are refined by the refinement modules which learn the DF maps.^41^ As opposed to,^41^ which uses it at the model’s final layer, multiple refinement modules are used in the decoder to enable the multi-scale feature maps to learn the DF maps. The strength of these multiscale refined feature maps is combined by feature aggregation, which is explained in the subsequent section. The combination of the features leads to more consistent feature refinement, resulting in a more accurate segmentation performance. The refinement module consists of a DF module and a feature rectification fusion (FRF) module. The DF map of the features is learned by passing the feature through the DF module. The learned DF map is then combined with the original feature maps by an FRF module. The DF for each pixel *p* in the domain of image ω can be obtained with the expression:


$$\text{DF}(p)=\{\begin{array}{cc} \frac{\vec{bp}}{\left|\vec{bp}\right|}, & \text{if}\;p\in \text{foreground},\\ \left(0,0\right) & \text{otherwise}, \end{array}$$

where *p* is the foreground pixel, and *b* is the pixel that is nearest to *p* lying on the boundaries of the vocal tract and articulators, while $\vec{bp}$ is a direction vector pointing from *b* to *p*. The pixels not belonging to the foreground are set to (0,0). The process is completed by introducing a DF module that consists of a 1 × 1 convolution layer, with the 2-channel DF as its output. The 2-channel DF specifically offers the direction and magnitude information of the DF.

In addition, the FRF module is used to incrementally correct the errors in the initial segmentation maps by utilizing information from the central region, and the direction information from the DF’s output. In other words, the initial feature maps input can be expressed as $F^{\left(0\right)}\in R^{C\times H\times W}$. By iteratively correcting the segmentation feature map, the feature map at step *k* can be expressed as below:


$$\forall p\in \omega ,F^{k}\left(p\right)=F^{\left(k-1\right)}\left(p_{x}+\text{DF}{\left(p\right)}_{x},p_{y}+\text{DF}{\left(p\right)}_{y}\right),$$

where k∈{1,N} denotes the current step, and *N* is the total steps, which is set to 6 for segmenting the speech MRI data. $p_{x}$ and $p_{y}$ represent the corresponding coordinates for the pixel *p*. Finally, $F^{k}$ is concatenated with $F^{0}$ to combine the direction information with the initial segmentation feature map and pass another 1 × 1 convolutional layer.

#### Feature aggregation

The final segmentation is obtained by first up-sampling all of the refined feature maps to the same shape as the decoder output, and then adding the up-sampled features to the decoder output.

## Experiment and results

This research used a publicly available dataset and was carried out as part of a research project approved by the UK Health Research Authority (IRAS 287981, February 3, 2022).

### Dataset

#### Speech MRI dataset

A publicly available dataset of midsagittal speech MRI series with corresponding GT segmentation and velopharyngeal closure labels was used.^42^^,^^43^ The dataset consists of 393 images (size 256 × 256 pixels) in 5 series of 105, 71, 71, 78, and 67 images. GT includes 6 classes: “head,” “soft palate,” “jaw,” “tongue,” “vocal tract,” and “tooth space.” Velopharyngeal closure labels are defined by contact between the postpharyngeal wall included in the “head” class and the “soft palate” class. As the dataset comprises 5 series exhibiting resemblances between images within each series, a 5-fold cross-validation was used. The velopharyngeal closure event analysis follows the methodology defined in,^24^ where they are classified as correct, additional, merged, or missed. Events are still classified as correct if their duration is extended by one frame at the start or the end of the closure.

##### Automatic cardiac diagnosis challenge dataset

The ACDC dataset^39^ was used to assess the generalizability of the proposed model on different type of MRI data. The dataset consists of 100 cardiac MRI scans from different patients with 3 classes; right ventricle (RV), spleen (SP), and myocardium (Myo). For our experiments, the dataset was split in the same way as MT-UNet,^44^ for which, 70 cases (1304 axial slices) are used for training, 10 cases (182 axial slices) are used for validation and 20 cases are used for testing.

### Training

**Figure 2.**
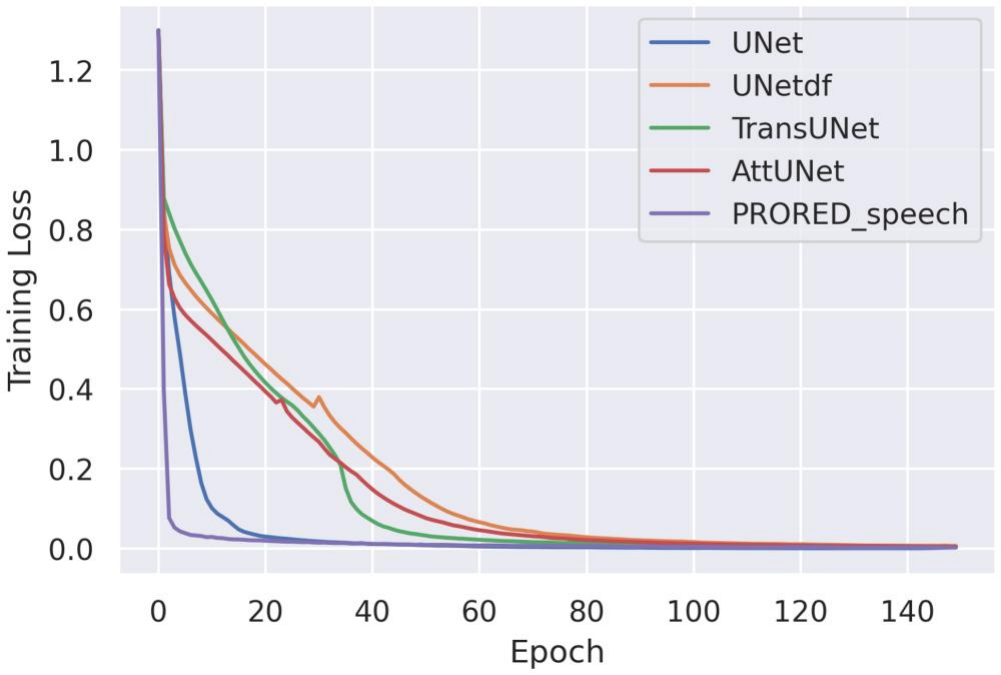
Training loss plots for the baseline and proposed models from one of the folds on speech MRI data.

**Figure 3.**
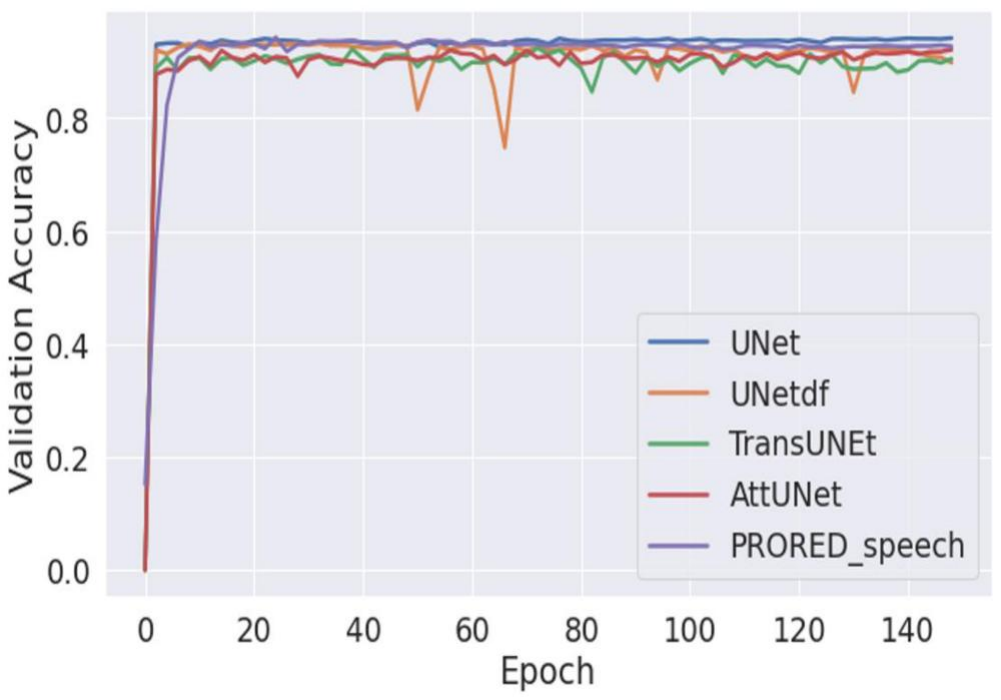
Validation accuracy plots for the baseline and proposed models from one of the folds on speech MRI data.

In addition to the proposed model, 4 different baseline models were implemented: UNet,^45^ Att-UNet,^46^ TransUNet,^40^] and Cheng et al.^41^ The later employs a UNet with a DF module as the post-processing step, it is referred to as UnetDF in the rest of the paper. Following Ruthven et al.,^24^ the UNet has channel numbers of 64,128,258,512,1024 for the encoder. For a fair performance comparison, all models were implemented on the same A5000 graphic card using Pytorch 1.10. Adam optimizer and Dice loss function were used and each model was trained for 150 epochs. It can be observed from Figures 2 and 3 that before reaching the maximum epoch number, all models’ training loss converge to zero and validation accuracy becomes stable. While the baseline models are publicly available, the default hyperparameters of them showing slower convergence. Therefore, we used learning rate of 0.0003 for training the baseline models and the best model is saved when the validation accuracy is the highest, and training loss has converged. Five-fold cross-validation was applied to all the models, ensuring that a different subject was tested in each fold. For each cross-validation fold, the data are divided into training, validation, and test sets, with 3 subjects allocated for training, 1 for validation, and 1 for testing. The training dataset is used to train the model. The validation dataset is used to evaluate the model’s performance during training. The test dataset is used to assess the model’s final performance. All reported results represent the average performance across 5-fold cross-validation on the test dataset. The hyperparameters for optimization were set to $\beta_{1}$ = 0.9, $\beta_{2}$ = 0.999, and ξ = 1$e^{-}8$. These 3 hyper-parameters are the same as the ones used in Ruthven et al.^24^ The training batch size was 8 and the learning rate was 0.0002 for the experiments. ResNet-50^47^ and ViT^33^ were combined in the hybrid encoder. The transformer backbones and ResNet-50 were pre-trained on ImageNet.

### Segmentation results

#### Speech MRI dataset

The performance of the different models over the 6 classes of the dataset is summarized in Table 1. All models were implemented 3 times and the average performance in dice score coefficient (%) is reported in the table. Our proposed model shows superior overall performance in comparison to the baseline models, with the best mean Dice score. The proposed model achieves the highest Dice scores for the “Jaw” and “Tongue” classes, with dice coefficients of 97.69% and 98.64%, respectively. Furthermore, the proposed model only differs from the best results of the baseline models in the remaining classes by a small margin. Specifically, TransUNet, Attention-UNet, UNetDF, and UNet achieved the highest dice coefficients in the head, soft-palate, tooth-space, and vocal-tract classes, respectively. The reason our method does not perform best in these classes could be that deploying multiple DF modules in the decoder may lead to over-smoothing of the segmentation. In addition, Table 2 presents the Hausdorff distance for the different networks. The proposed model greatly improves the segmentation accuracy in the tooth space, tongue and soft palate. The latter 2 are of particular clinical importance, with improved delineation of the soft palate being crucial to the study of velopharyngeal closure (see further down). The soft palate and the tooth space are the 2 smallest classes, thus the good results on these 2 classes highlight the strength of the proposed network in learning fine features.

**Table 1.** Overall and classes segmentation performance (Dice coefficient, %) for the different models with bold values being the highest Dice coefficient.

Model	Head	Jaw	Soft palate	Tongue	Tooth space	Vocal tract	Param	Mean dice
UNet^45^	99.02	96.86	96.66	98.23	96.03	**97.05**	31.05M	97.31± 0.05
Att-UNet^46^	99.17	96.73	**97.35**	97.84	97.00	96.74	34.88M	97.47 ± 0.04
TransUNet^40^	**99.46**	96.65	96.66	98.31	95.53	96.70	104.10M	97.22 ± 0.04
UNetDF^41^	99.18	97.48	97.21	98.43	**97.08**	96.41	34.53M	97.63 ± 0.04
Proposed	99.44	**97.69**	97.29	**98.64**	96.65	96.87	104.97M	**97.78** ± **0.03**

**Table 2.** Overall and classes segmentation performance (Hausdorff distance, mm) for the different models with bold values being the lowest Hausdorff distance.

Model	Head	Jaw	Soft palate	Tongue	Tooth space	Vocal tract	Mean HD
UNet^45^	18.77	9.36	6.37	12.09	4.38	21.89	12.14 ± 22.16
Att-UNet^46^	**9.52**	8.54	2.29	6.28	2.39	**6.82**	**5.97** ± **10.77**
TransUNet^40^	16.34	7.66	3.60	18.65	2.75	14	10.84 ± 20.95
UNetDF^41^	12.27	**4.4**	3.18	4.43	3.33	10.47	6.35 ± 11.83
Proposed	19.1	6.14	**1.54**	**4.32**	**1.92**	8.01	6.84 ± 13.62

**Figure 4.**
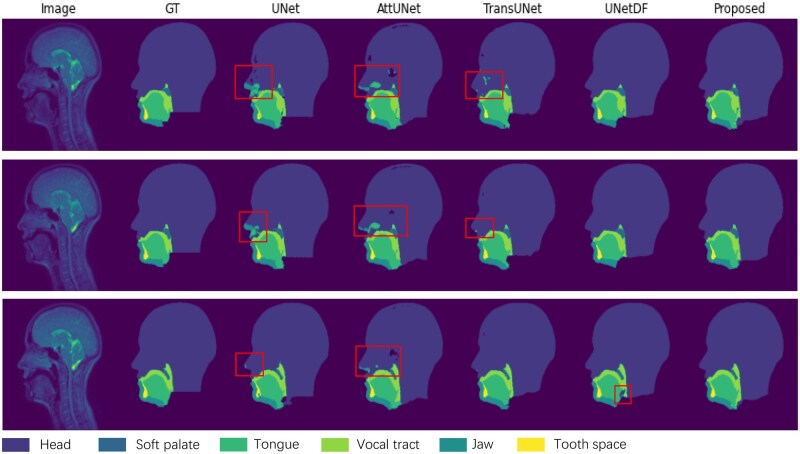
Comparison of examples of GT segmentations and outputs of the different networks on the speech MRI dataset. From left to right: speech MRI image, GT, segmentation of UNet, Attention UNet (Att-Unet), TransUNet, UNet with DF module and the proposed model. The red rectangles highlight areas where the “head” class is being misclassified.

Figure 4 shows the qualitative performance comparing the proposed model with 3 baseline models: UNet, Att-UNet, and TransUNet. It can be observed that class confusion, where parts of the head are misclassified due to similar contrast to other classes, especially around the nose and hard palate, is greatly reduced in the proposed model. The red rectangles on Figure 4 indicates the area where head is head region is misclassified. Misclassification in those areas is more prevalent with UNet and Att-UNet.

#### ACDC

**Figure 5.**
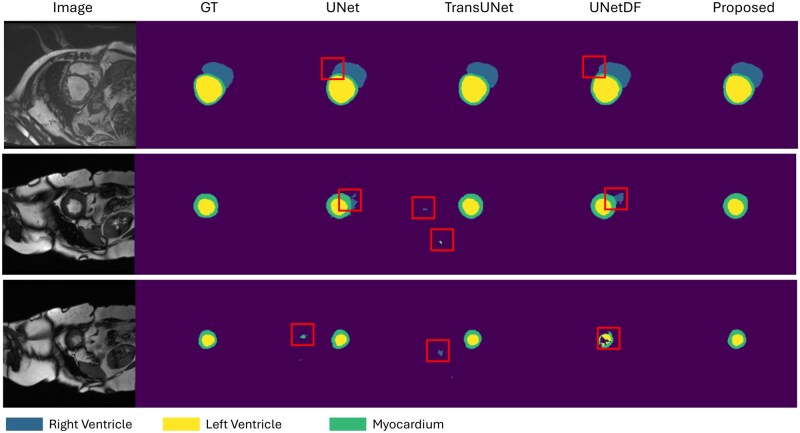
Comparison of the segmentation performance on ACDC dataset. From left to right are the image, GT, segmentation of UNet, TransUNet, and the proposed PRORED model. The red rectangles highlight areas where there are misclassified pixels.

In addition to segmenting the speech MRI dataset, we evaluate the model on the ACDC dataset to ensure the generalizability of the proposed approach. The quantitative performance of the models on the ACDC dataset is presented in Table 3. Note that the performance of UNet on the ACDC dataset reported in the table is based on the results reported by Feng et al.^41^ We compare the proposed model with UNet with DF and TransUNet. Our results show that we achieve the highest average Dice coefficient, with 2 out of 3 classes outperforming the baseline models. Additionally, replacing the cascaded decoder with PRORED improves the Dice coefficient by 2.2%, highlighting the effectiveness of PRORED. As shown in Figure 5, the proposed model results in fewer misclassified pixels compared to the baseline models. Additionally, the first row of the comparison illustrates that the proposed model better preserves the shape of the right ventricle.

**Table 3.** Segmentation performance of the proposed model with TransUNet and UNet with DF module on ACDC with bold values being the highest Dice coefficient.

Model	RV	Myo	LV	Avg Dice
UNet^41^	85.60	87.20	93.10	88.60
TransUNet^40^	86.67	87.27	95.18	89.71
UNetDF^41^	**91.10**	88.80	94.90	91.60
Proposed	90.64	**89.56**	**95.50**	**91.90**

### Closure pattern analysis

**Figure 6.**
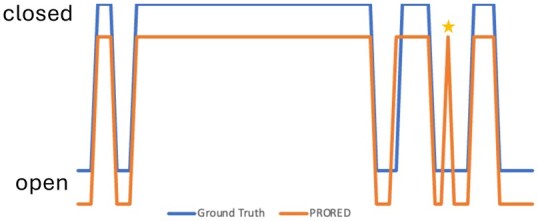
Comparison of closure pattern between the GT (blue) and PRORED (orange) in subject 2. The asterisk marks a false closure event prediction by PRORED. The third closure event starts one frame early for PRORED.

**Figure 7.**
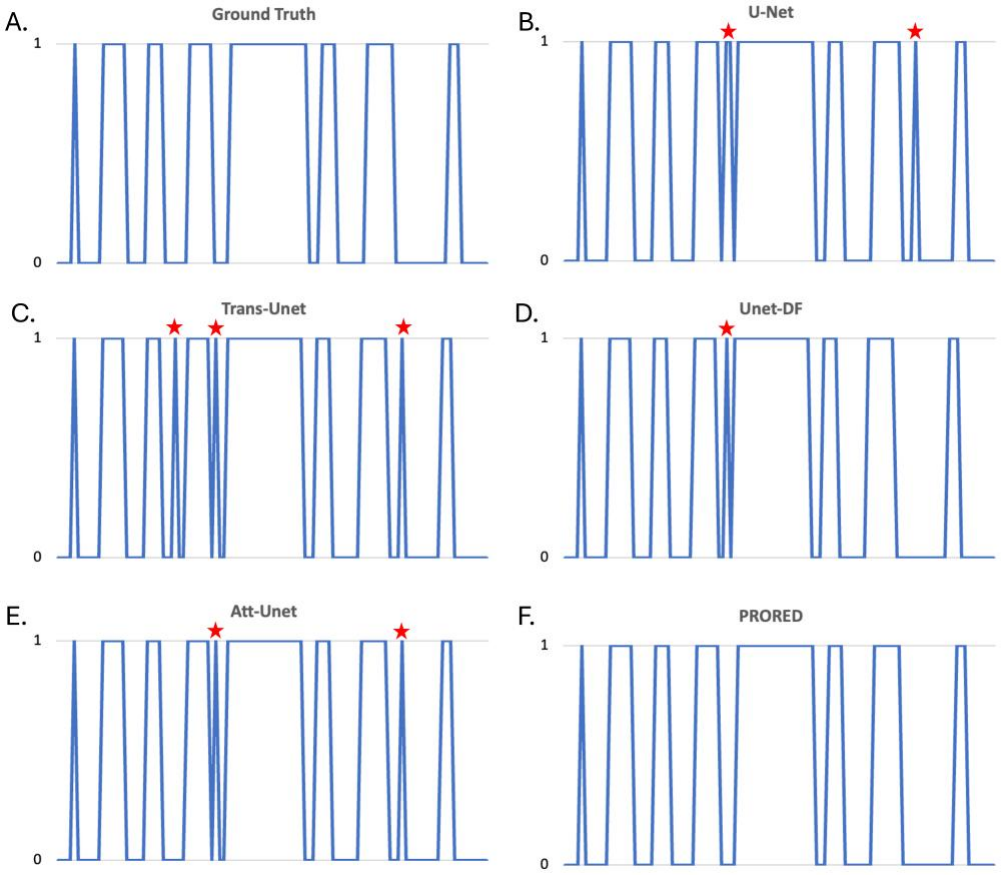
Comparisons of closure patterns for all networks in the subject with the lowest image quality (subject 1). 0 = velopharyngeal port open, 1 = closed, red asterisks mark a false closure event prediction. All network bar our proposed model (PRORED) led to 1 to 3 false closure events in this subject.

The correct classification of individual frames with regards to velopharyngeal closure, as defined in the publicly available dataset,^42^ is given in Table 4. The model we propose achieved the best accuracy rates of all models with an accuracy nearing 99%, closely followed by UNetDF.^41^ Our model had the lowest rate of false positive “closed” frame and no false negative. When performing a closure event analysis (Table 5) using the methodology described by Ruthven et al.,^24^ our model also outperforms all other models with all closure events identified correctly and only one additional closure event, this is illustrated in Figure 6. This extra closure event was created by all the networks tested and correspond to some saliva effectively joining the uvula and the post-pharyngeal wall in a single frame leading to a false closure event. For all models, the vast majority of misclassified frames are at the end or beginning of a closure events as can be seen in the 2 curves not fully overlapping in Figure 6. Those are images where the soft palate is in very close proximity to the post-pharyngeal wall. When looking at closure patterns, UNetDF^41^ was once again the second best performing network with 2 extra closure events. This additional one is highlighted in Figure 7, where all models, bar the one we proposed, created 1 to 3 additional closure events. This subject has overall a poorer image quality. Those results, combined with lower Hausdorff distance for the soft palate (Table 2) indicate that our network is better at delineating this organ. The proposed network also improves the segmentation of the second smallest class (tooth space). It might be worth noting that all the networks we implemented, including our own version of Unet, have a better performance than the Unet model proposed by Ruthven et al.^24^ (Table 5) and unlike their models do not create merged closure events when 2 or more events are detected as a single one. The main difference in the 2 Unet implementations is that Ruthven et al.^24^ used optimizer learning rates of 0.001 and 0.0001, whereas the learning rate in our implementation was 0.0003 leading to a faster training loss convergence.

**Table 4.** Individual frames classification accuracy for velopharyngeal closure, including false positive and false negative (%) with bold values indicate the best results among models.

	**UNet** ^45^	**AttUNet** ^46^	**TransUNet** ^40^	**UNetDF** ^41^	Proposed
Accuracy	96.68	97.19	97.45	98.48	**98.98**
False positive	2.55	2.81	2.29	1.52	**1.02**
False negative	0.77	**0.00**	0.32	**0.00**	**0.00**

**Table 5.** Closure event analysis comparison.

	Total	Correct	Additional	Merged	Missed
GT	30	30	n/a	n/a	n/a
Ruthven et al.^24^	33	27	5	3	0
UNet^45^	33	30	3	0	0
AttUNet^46^	33	30	3	0	0
TransUNet^40^	34	30	4	0	0
UNetDF^41^	32	30	2	0	0
Proposed	31	30	1	0	0

N/A indicates that closure/open parameters are not applicable to the ground truth, as it represents the fixed standard against which the models are evaluated.

### Ablation study

To demonstrate the effectiveness of the model, an ablation study was carried out on the number of refinement modules. The ablation study results are presented in Table 6. The model without and with one refinement module achieves mean dice of 97% and 97.35%, respectively. It indicates that adding the refinement module to the model improves the model performance. Furthermore, Due to the over-smooth of the feature classes’ edges, adding more refinement modules to the model does not always improve the model’s performance. The model with 3 refinement modules achieved the best performance in terms of mean dice. This pattern, where the inclusion of 3 refinement modules in the decoder yields the best performance, was observed across all 3 runs of the repeated experiments.

**Table 6.** Ablation study on the number of refinement modules in PRORED with mean dice coefficient reported in percentage (%).

No. of ref	0	1	2	3	4
Speech	97.00	97.35	97.74	97.78	97.44
ACDC	91.20	91.49	91.62	91.90	91.53

## Discussion

In this paper, an effective network for automatic segmentation of the vocal tract and articulators in dynamic speech MR images is proposed. The proposed model features a hybrid CNN-transformer encoder that captures both local and global contexts within features. It is paired with a novel decoder, PRORED, which progressively refines and upsamples features at multiple scales. Testing on a widely used speech MRI dataset demonstrated that this network is highly effective at segmenting articulators and vocal tracts in speech MRI images, achieving an impressive average Dice coefficient of 97.78%. Additionally, by analysing open and closure patterns in the segmented masks produced by our network, we attained a classification accuracy of 94.67%. The high accuracy in identifying closure patterns in speech MRI is crucial for developing an automated tool for diagnosing VPI. The accurate segmentation and classification performance of PRORED confirms its potential as a reliable support tool in the clinical diagnosis of VPI. Nevertheless, using a hybrid encoder comes with extra computational costs; in this model, the hybrid encoder uses a combination of residual encoder and vision transformer encoder both are pre-trained on Imagenet, thus adding to the computational cost to the network and therefore, increasing the time it takes to train the model. The proposed network took 2.2 h for training, while UNet took 1.3 h. For clinical applications, efficiency is crucial. To lessen the computational load, network structure optimization will be a major direction for future work. In our current experiments, the hybrid encoder is responsible for the majority of the computational cost. Given that our proposed decoder network can be easily integrated with various encoders, other lighter encoders, such as swiftformer^48^ and pyramid vision transformer,^49^ convnet^50^ could be considered as a potential replacement for the hybrid encoder to reduce the computation cost.

Despite achieving high accuracy, the task of manually labelling segmented maps for classification remains labour intensive and time consuming. Recognizing this limitation, the next phase of research is dedicated to developing a model capable of automatically classifying closure patterns directly from segmented maps. This innovative step is expected to significantly streamline the segmentation and classification process, enhancing efficiency and reducing the dependency on manual labelling efforts. By automating this crucial step, we aim to expedite the diagnostic process for VPI, ensuring quicker and more accessible clinical assessments. The advancement not only promises to improve the workflow for researchers and clinicians but also sets the stage for further innovations in automated speech MR image analysis.

## Conclusion

The study presents a novel decoder architecture tailored for speech MRI dataset segmentation called PRORED. The model addresses the need for a method capable of handling the complex structures within speech MRI data. A key feature of PRORED is its capability to learn DF maps during the up-sampling process. This capability is crucial for accurately restoring segmented images with precise boundaries between different classes. This allows for better segmentation of smaller classes and in particular the soft palate, thus improving automatic velopharyngeal closure analysis. However, the multiple DF modules deployed in the decoder could result in over-smoothing of the feature maps. In our future work, we plan to improve the model by conditionally refining features based on their semantics. This approach could mitigate the issue of over-smoothing and improve overall performance.
